# First case of pregnancy and childbirth in a patient implanted with an extravascular implantable cardioverter-defibrillator

**DOI:** 10.1016/j.hrcr.2025.10.044

**Published:** 2025-11-04

**Authors:** Kirsten M. Kooiman, Jolien A. de Veld, Willeke van der Stuijt, Reinoud E. Knops

**Affiliations:** 1 Amsterdam UMC location University of Amsterdam, Heart Center, Department of Cardiology, Amsterdam Cardiovascular Sciences Heart failure & Arrhythmias, Amsterdam, The Netherlands

**Keywords:** Extravascular ICD, Pregnancy, Childbirth, Ventricular arrhythmia, Oversensing

**Key Teaching Points**

• During pregnancy, the sensing signal from an extravascular implantable cardioverter-defibrillator may change, owing to rotation of the heart. Therefore, recurrent monitoring is advised.
• In case of over- or undersensing, the implantable cardioverter-defibrillator settings may be adjusted during pregnancy and reprogrammed after birth.
• We reported no device-related complications during pregnancy and childbirth.

## Introduction

The extravascular implantable cardioverter-defibrillator (EV-ICD) was developed as an extravascular alternative to the transvenous ICD (TV-ICD).^1^ In contrast to the already commercially available subcutaneous ICD (S-ICD), the lead of the EV-ICD is implanted in the substernal space, enabling it to deliver antitachycardia and pause-prevention pacing.^2^ Owing to the close proximity to the heart, the EV-ICD has a proven lower defibrillation threshold (DFT) than the S-ICD, and therefore, the generator size is comparable with a transvenous device, with an output of 40 J.^2^^,^^3^ This feature makes the EV-ICD a desirable extravascular alterative for slim female patients, given that these patients may experience issues with the larger S-ICD generator.^4^

With the increased recognition of genetic arrhythmia syndromes, the age at which some patients receive an ICD declines. Because of this, there is an increase in female patients who become pregnant with an ICD in situ. Several small studies have been performed on pregnancy and childbirth with a TV-ICD or S-ICD.^5^, ^6^, ^7^, ^8^, ^9^ These studies showed few complications or inappropriate ICD discharges, and therefore, treatment with an ICD during pregnancy is considered safe.^10^ However, given that the EV-ICD is a new device, no data have been published on pregnancy with an EV-ICD. The EV-ICD is a completely extracardiac device, but the lead is implanted intrathoracically below the sternum. During pregnancy, the position of the heart in the thorax changes and there is a decrease in the intrathoracic space, which might have an effect on sensing. In this report, we present the first case of pregnancy and childbirth in a patient implanted with an EV-ICD.

## Case

**Figure 1:**
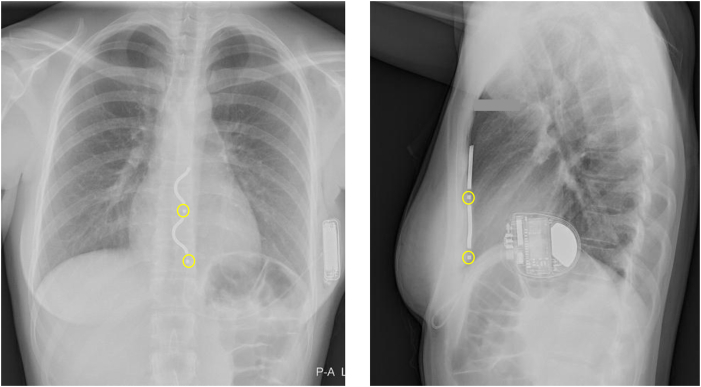
EV-ICD implantation position. EV-ICD position right after implantation. The *yellow circles* indicate the sensing electrodes. EV-ICD = extravascular implantable cardioverter-defibrillator.

**Figure 2:**
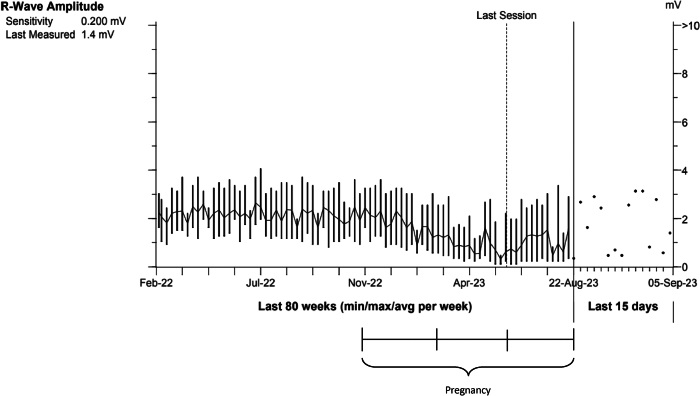
R-wave amplitude. The figure shows a decrease in R-wave amplitude during pregnancy. The *black line* below indicates the pregnancy duration divided into trimesters.

**Figure 3:**
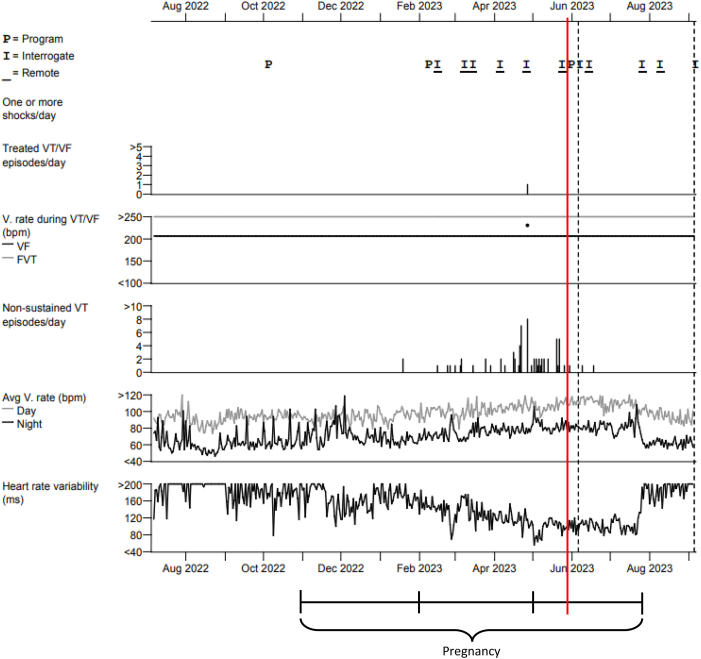
Cardiac compass. The figure shows an increase in oversensed episodes during pregnancy and a decrease in heart rate variability. The *black line* below indicates the pregnancy duration divided into trimesters. The *red line* indicates the moment of change in settings. FVT = fast ventricular tachycardia; VF = ventricular fibrillation; VT = ventricular tachycardia.

**Figure 4:**
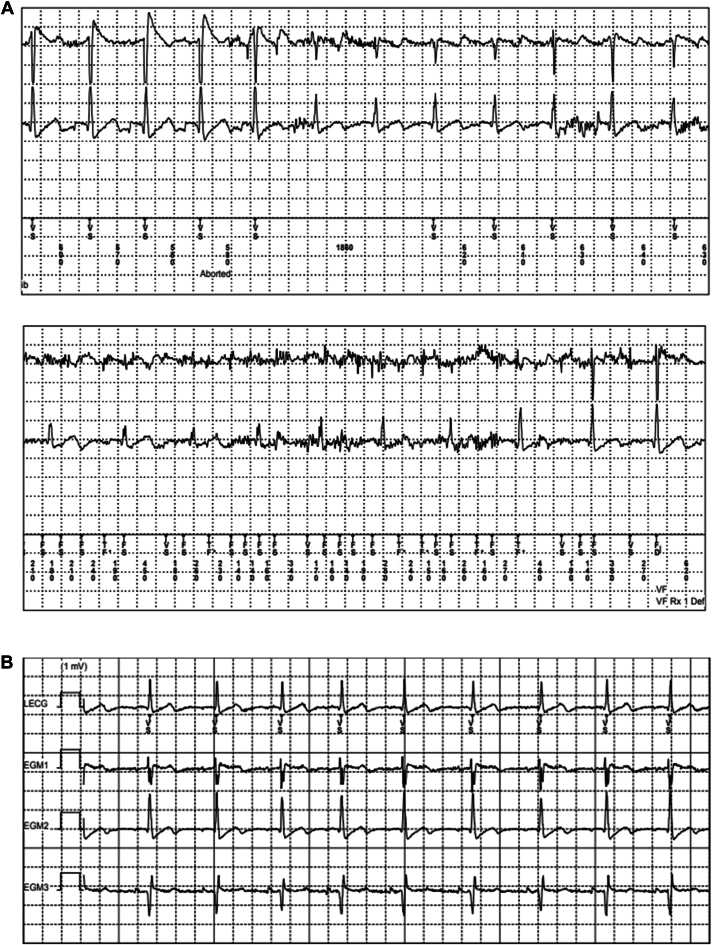
**A:** Oversensing owing to a small R-wave amplitude leading an aborted shock. From top to bottom: EGM 1, ring1–ring2; EGM 2, coil 1 to can; markers, V–V (ms). **B:** EGM before pregnancy. EGM = electrogram.

A 24-year-old female patient (sex assigned at birth) received an EV-ICD for primary prevention after she was diagnosed as having an increased risk of idiopathic ventricular fibrillation (VF) owing to a genetic mutation in chromosome 7. She had no comorbidities. The postoperative chest radiograph is presented in Figure 1. After implantation, she had a successful DFT at 20 and 15 J at a programmed sensitivity of 0.45 mV. After 2 years of follow-up, she became pregnant with her first child. Until her pregnancy, the patient had not experienced any appropriate or inappropriate therapy from her ICD and only incidental registration of short oversensing episodes occurred, with the oversensing prevention setting already programmed on 6 (nominal medium −3). At that time, her pause-prevention alerts were set at 1, the sensitivity at 0.150 mV, and detection at 30/40. The patient started with remote monitoring when she was 14 weeks pregnant. At 19 weeks of pregnancy, we noticed a change in the R-wave amplitude signal of the EV-ICD. The signal had decreased from 2 mV to 0.6 mV (Figure 2), which resulted in multiple episodes of oversensing (Figure 3). Initially, the remote transmission frequency was increased, and no changes were made to the ICD sensing and detection settings. After 6 months of pregnancy, more oversensing episodes were stored as nonsustained ventricular tachycardia (VT). In addition, the oversensing even led to an aborted shock (Figure 4). Hereafter, the EV-ICD settings were adjusted. The sensitivity was set to 0.2 mV, initial detection was extended to 45/60, and the pause-prevention alert was changed from 1 to 3. Even though this led to some undersensing and pause-prevention alerts, this eliminated the oversensing and prevented inappropriate ICD discharges. Apart from these sensing issues, the EV-ICD demonstrated a decrease in heart rate variability (HRV) during the progression of the pregnancy, which is also shown in Figure 3. At last, the patient did not experience any ventricular arrhythmias during pregnancy.

During childbirth, the EV-ICD shock function was deactivated using a magnet. Six weeks after childbirth, her EV-ICD signal increased again, and the settings were set back to her initial situation. The sensitivity was lowered to 0.15 mV, detection was set at 30/40, and pause prevention was changed back from 3 to 1. Follow-up to date shows incidental storage of oversensing episodes categorized as nonsustained VT, which are similar to those experienced before the pregnancy.

## Discussion

To the best of our knowledge, this is the only case reported of a pregnant EV-ICD patient. In the EV-ICD pivotal study, current or expected pregnancy was an exclusion criterion, and therefore, little is known about the devices’ functionality under these circumstances. However, given that the EV-ICD is an especially suitable alternative for young patients in whom avoidance of leads in the vasculature is valued, there is a need for knowledge about pregnancy with this device.

The patient presented in this case report experienced an increase in oversensing episodes during pregnancy, owing to a decrease in the R-wave amplitude signal. During fetal growth, the uterus pushes the diaphragm upwards, resulting in a rotation of the heart to a more horizontal position.^11^ Given that the EV-ICD lead remains in the same position, this cardiac rotation may result in an altered sensing electrogram and lower R wave in the programmed sensing vector (usually ring1–ring2). The sensing threshold after a sensed R wave is based on a percentage of the R wave, so if the amplitude of this wave decreases, the sensing threshold is lowered and becomes more susceptible to oversensing. An analysis by Swerdlow et al^12^ also found that a decrease in R wave is associated with more P wave and myopotential oversensing, which are the most common causes of inappropriate sensing in the EV-ICD. After the aborted shock and increase in oversensing events in our patient, the sensitivity was increased and detection was extended to 45/60, to lower the risk of oversensing of T waves and noise owing to a smaller R/T ratio. With adjustments in the sensitivity of the EV-ICD, the risk of undersensing increases, and therefore, it is recommended by the manufacturer to maintain a safety margin for detecting VF of at least 3 times the final programmed sensitivity. Failing to include this safety margin may result in undersensing of VT/VF, which must be avoided, particularly because physiological changes and heightened sympathetic activity during pregnancy can precipitate ventricular arrhythmias.^13^ For patients in whom adequate sensing cannot be achieved and performing a DFT would be favorable to evaluate adequate sensing of VT/VF, deactivation of the EV-ICD and temporary use of a life vest until the R-wave signal improves may be considered as an alternative solution.

The reason why we do not recommend immediate alterations in the EV-ICD sensitivity at the beginning of pregnancy is that it is unknown whether cardiac rotation always results in a decrease in the signal or can also result in an increase. Therefore, incrementing the monitoring frequency to see how the cardiac signal develops would be a more appropriate strategy.

Oversensing is more common among extravascular and S-ICD carriers than TV-ICD patients. The TV-ICD registers an intracardiac signal from the specific implantation location of the lead, whereas the EV-ICD and S-ICD are more susceptible to oversensing of P and T waves and noise from noncardiac stimuli. Therefore, oversensing is generally a normal consequence of how extracardiac devices sense the activity of the heart. Although transvenous devices rely on beat-to-beat detection, extracardiac devices are designed to detect overall rhythm patterns. However, inappropriate ICD shocks are associated with a significant reduction in quality of life, and therefore, long-lasting episodes of oversensing should be prevented.^14^ During pregnancy, this can be done by increasing the follow-up frequency to evaluate sensing, as was done in our case.

### HRV

HRV reflects changes in the autonomic nervous system, indicating shifts between sympathetic and parasympathetic activities. Low HRV is associated with an increased risk of all-cause mortality, and low HRV has been proposed as a marker for disease.^15^ Abnormal HRV patterns may help predict pregnancy complications such as preeclampsia.^16^ As expected during pregnancy, the HRV of our patient decreased.^17^ Although the use of HRV measurements in pregnant women with an EV-ICD is unexplored, it may offer meaningful insights into physiological changes over the course of pregnancy.

### ICDs during childbirth

In our patient, the shock function of the EV-ICD was deactivated during childbirth, owing to the lack of experience with this device during vaginal delivery. Deactivation of the ICD comes with logistical challenges. The ICD can be deactivated through programming by the ICD technician or by placing a magnet on the ICD when the technician is not available, for example, when labor initiates outside office hours. After deactivation of the ICD, the patient often requires continuous monitoring of the cardiac rhythm, restricting movement. Earlier research regarding the safety of an activated ICD during childbirth has not shown any complications or inappropriate ICD discharges, but these data are limited to abdominal, transvenous, and subcutaneous devices.^5^ In a recent position statement, it was recommended to keep the ICD in full therapy mode during childbirth, with a magnet available if required.^18^ This approach could also be considered during childbirth in patients with an EV-ICD, although further data are needed to support and validate this strategy.

## Conclusion

During pregnancy, the sensing of the EV-ICD may change owing to rotation of the heart, which could lead to inadequate sensing. This can result in an increase in oversensing episodes and potentially inappropriate device discharges. Therefore, it is recommended to frequently monitor the patient and adjust sensing if necessary. Additional cases are needed to provide recommendations for EV-ICD management during childbirth.

## Disclosures

K.M.K. reports consultancy fees from Boston Scientific. R.E.K reports consultancy fees and research grants from Abbott, Boston Scientific, Medtronic, and Cairdac and has stock options from AtaCor Medical Inc. J.A.d.V. and W.v.d.S. declare no conflicts of interest.
